# A unified approach of detecting phase transition in time-varying complex networks

**DOI:** 10.1038/s41598-023-44791-3

**Published:** 2023-10-20

**Authors:** Mohamed Ridha Znaidi, Jayson Sia, Scott Ronquist, Indika Rajapakse, Edmond Jonckheere, Paul Bogdan

**Affiliations:** 1 Ming Hsieh Department of Electrical Engineering, University of Southern California, Los Angeles, CA 90089 USA; 2 Department of Computational Medicine and Bioinformatics, University of Michigan, Ann Arbor, MI 48109 USA; 3 Department of Mathematics, University of Michigan, Ann Arbor, MI 48109 USA

**Keywords:** Computational biology and bioinformatics, Engineering

## Abstract

Deciphering the non-trivial interactions and mechanisms driving the evolution of time-varying complex networks (TVCNs) plays a crucial role in designing optimal control strategies for such networks or enhancing their causal predictive capabilities. In this paper, we advance the science of TVCNs by providing a mathematical framework through which we can gauge how local changes within a complex weighted network affect its global properties. More precisely, we focus on unraveling unknown geometric properties of a network and determine its implications on detecting phase transitions within the dynamics of a TVCN. In this vein, we aim at elaborating a novel and unified approach that can be used to depict the relationship between local interactions in a complex network and its global kinetics. We propose a geometric-inspired framework to characterize the network’s state and detect a phase transition between different states, to infer the TVCN’s dynamics. A phase of a TVCN is determined by its Forman–Ricci curvature property. Numerical experiments show the usefulness of the proposed curvature formalism to detect the transition between phases within artificially generated networks. Furthermore, we demonstrate the effectiveness of the proposed framework in identifying the phase transition phenomena governing the training and learning processes of artificial neural networks. Moreover, we exploit this approach to investigate the phase transition phenomena in cellular re-programming by interpreting the dynamics of Hi-C matrices as TVCNs and observing singularity trends in the curvature network entropy. Finally, we demonstrate that this curvature formalism can detect a political change. Specifically, our framework can be applied to the US Senate data to detect a political change in the United States of America after the 1994 election, as discussed by political scientists.

## Introduction

Driven by the implications of complex networks (CNs) on many applications, studying CNs has become of crucial importance for designing efficient algorithms to control or predict the behavior of such networks. In plenty of real-world applications, CNs are not static but vary in time. For example, these applications range from genetic interactions in the human genome, microbial to neuronal interactions in the gut-brain axis, to spread of epidemic disease on social networks^1^. A practical mathematical model of such networks is to represent them as time-varying graphs, where the interactions between different entities (nodes) are generally marked as links (edges) with the strength of the interactions encoded as weights. For instance, in social networks, an edge between two nodes means that there exists a relationship between them, and the weight on that edge encodes the strength of similarity or measure of influence. It follows that adding or strengthening interactions between entities in a CN has a direct effect on the state of the network under study. More specifically, while the network is evolving in time, either by introducing new links or modifying existing weights, the topological properties of the CN may change drastically which we characterize as a network phase transition. Therefore, the investigation of the time evolution of these local interactions is a key step in understanding the causal predictive power of a network.

Consider a CN (having the same set of nodes) in two different states where this difference can be a consequence of a change in its topological properties. One can treat them analogously without the knowledge of the two states. That is why it is imperative to propose an approach to quantify the state of a given network in order to analyze it correctly. A similar setup can also be depicted for studying TVCNs where a phase is characterized by invariant network characteristics over a time interval. For instance, the time-varying interactions can drive the network from one phase to another (such as the emergence of a self-optimization behavior of the network information transfer observed in brain-derived neuronal networks^2^). The objective is to decipher non-trivial local properties of a network and how these affect its global behavior.

Although, the problem of detecting such a change has attracted the interest of many researchers, it has not been extensively studied in previous work especially for weighted CNs.

A framework to estimate change point in time-evolving networks, more specifically, in a sequence of evolving Markov random fields was proposed in Ref.^3^ where they suggested and investigated the statistical properties of a maximum penalized pseudo-likelihood estimate. Other works such as in Refs.^4^ and^5^ rely on the underlying network generating model to perform a maximum likelihood based approach to identify change points. The analysis of small-world networks has been performed based on computation of clustering coefficient and average path length in Ref.^6^ to prove the existence of a phase transition which occurs depending on the rewiring probability parameter. Along the same lines, an alternative approach focused on studying random growing networks^7^ where it claimed that the change in degree distribution depends on the attachment probability parameter. Finally in Ref.^8^, the phase transition detection is based on the core community structure recovery.

In this context, identifying the change between different states of the network (i.e., phase transition) needs more sophisticated tools that go beyond the classical ones used for analyzing networks (e.g., degree distribution, average path length, modularity, assortativity, clustering, etc.) and being able to detect change points from as few snapshots of the TVCNs as possible.

On the other hand, geometric curvature analysis of networks has been recently advocated as a promising candidate for network analysis. Similar to curvature in differential geometry, where we use curvature to characterize an object by measuring its deviation from being flat, prior work^9–21^ analyzed the characteristics of a CN by looking at its curvature. Specifically, Ricci curvature (RC) has been introduced to analyze CNs in order to provide better intuition about the topology of CNs. In general, RC measures the deviation of a volume of a small geodesic ball in a curved Riemannian manifold from being a standard ball in Euclidean space^9,22–24^, hence, it measures the growth of geodesics’ volumes. In order to provide a more comprehensive topological characterization of TVCNs, the RC can identify the higher-order connectivity structure between different agents in a network; it offers mathematical tools for a better understanding of the non-trivial topological properties of a network across multiple scales. Along these lines, Ollivier proposed a discrete version of RC, (i.e., Ollivier-RC) that is related to optimal transport theory and allows to investigate discrete spaces like graphs^10,11,25,26^. For instance, the Ollivier-RC proved crucial to discovering the community structure in weighted and unweighted CNs^18,27^. Inspired by Bochner–Laplace operator^28^, Forman provided a second discrete version of RC for network analysis, (i.e., Forman-RC) which proves appealing for the analysis of large CN due to the computational advantages provided by its mathematical formula to estimate the localized curvature for each edge. Also, it is worth mentioning that Ollivier-RC directly captures the growth of volumes property of RC, while Forman-RC encodes the dispersion of geodesics property of RC. Recent works applied these two definitions in different scenarios to analyze networks^10–17^. In contrast to prior work, we focus on exploiting the Forman-RC concept to uncover the hidden properties of time-varying CNs which may inform us about the structural changes or anomalies that may occur.

To the best of our knowledge, detecting a transition from one state to another for TVCNs via a geometric based approach has not been considered before. Relying on the Forman-RC concept, we develop a novel algorithm for characterizing and detecting a significant change in the network’s structure. In contrast to prior work^3–8^ which focus on specific network models, community structure algorithmic strategies or other classical network metrics, a key ingredient to fully understand the time evolution of CNs is to analyze how scale-dependent interactions between different CN components (i.e., encoded in the weights of the graphs) contribute to localized geometric changes in the graph curvature and emerging phase transitions over time. The latter critical point has not been considered in most of the previous works. Taking advantage of the geometric analysis of the network’s structure, our work can take into account the aforementioned issue; also it can be applied not only to unweighted networks but also to weighted networks. This approach provides a more in-depth understanding of the time evolution of a CN; hence this formalism can be exploited for developing optimal control schemes required to bring one network under study from one phase to another.

The outline of the paper is as follows. First, we provide the results of our work applied to artificiality generated time-varying networks as well as neural networks. Then, experimental results for real-world dataset are provided to validate the presented framework. Next, we consider both the human fibroblasts proliferation dataset and the roll call votes of the United States Senate, and we map their dynamics onto the formalism of TVCNs. Applying our method, we retrieve the political change after the 1994 election in the USA, marked with the end of the “Conservative Coalition” according to political science. Finally, in the “Methods” section we present the mathematical model used for developing the proposed framework.

## Results

In this work, we consider TVCNs in which the nodes are fixed but with edge weights changing over time. Our objective is to be able to provide a multi-scale mathematical framework that can address the following questions: When does the topology of a complex network change? What are the different states of the complex network? To answer these questions and validate the proposed strategy, we proceed as follows: First, we generate artificial complex weighted networks according to a knob variable that can control whether the CN is approaching a random or a scale free graph and study how the Forman-RC based algorithm can identify the phase transition as a function of network sizes. Second, we use the rewiring probability to interpolate between a regular, a small-world and a random graph and demonstrate how the Forman-RC based algorithm can detect the phase transition.

Third, we mimic the phase transition as changes to the network community structure generated from a stochastic block model and observe the detecting capability of the Forman-RC based algorithm. Finally, we apply the proposed formalism to two real-world TVCNs (e.g., TVCNs corresponding to cellular reprogramming and political decision making). In all cases, this framework shows interesting results in the sense that it can detect the phase transition accurately.

**Figure 1:**
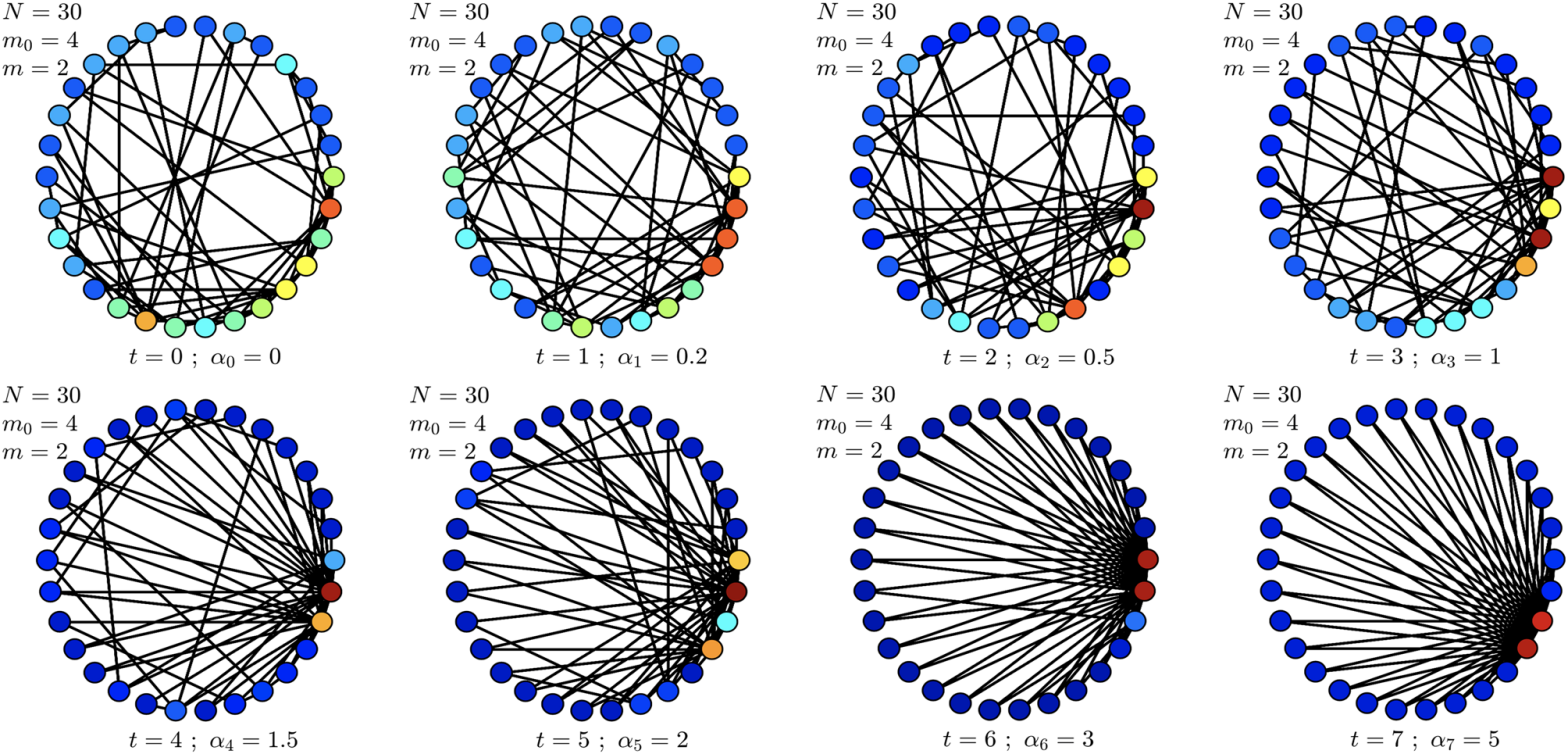
A sample time-varying network generated according to the random growing weighted network model. Each time *t* corresponds to a parameter $\alpha _t$ which is used to generate the network $G_t$ containing $N=30$ nodes and having parameters $m_0 = 4$ and $m=2$ (these parameters are similar to the ones in the Barábasi–Albert network model). Node colors are indicative of the node’s degree.

### Random growing weighted networks

To investigate the implications of curvature analysis on detecting phase transition phenomena, we first consider a random growing network obeying the Barabási-Albert inspired network model^29^. In order to be able to interpolate between different classes of networks (network phases) from Erdos–Renyi to scale-free and beyond, we modify the connection kernel, thus, the preferential attachment mechanism. Specifically, we generate a weighted network in which the preferential attachment probability for a node *i* in the network is given by the parameterized preferential probability $p_i=k^{\alpha }_i/\sum _{j}k^{\alpha }_j$, where $k_i$ is the degree of node *i* and $\alpha \ge 0$. Our intuition is that the parameterized preferential attachment mechanism plays an important role in defining the structure and the high-order topological properties of the Barabási-Albert network. Figure 1 illustrates a TVCN sample generated according to the random growing weighted network model for $N=30$ defined with $m_0 = 4$ and $m=2$. The presented 8 network snapshots in Fig. 1 corresponding to several parameter values for $\alpha _t$ (i.e., $\alpha _t = 0, 0.2, 0.5, 1, 1.5, 2, 3,$ and 5, respectively) which contribute to network topologies that interpolate between a random network and a more regular one (despite the networks are being generated randomly).

**Figure 2:**
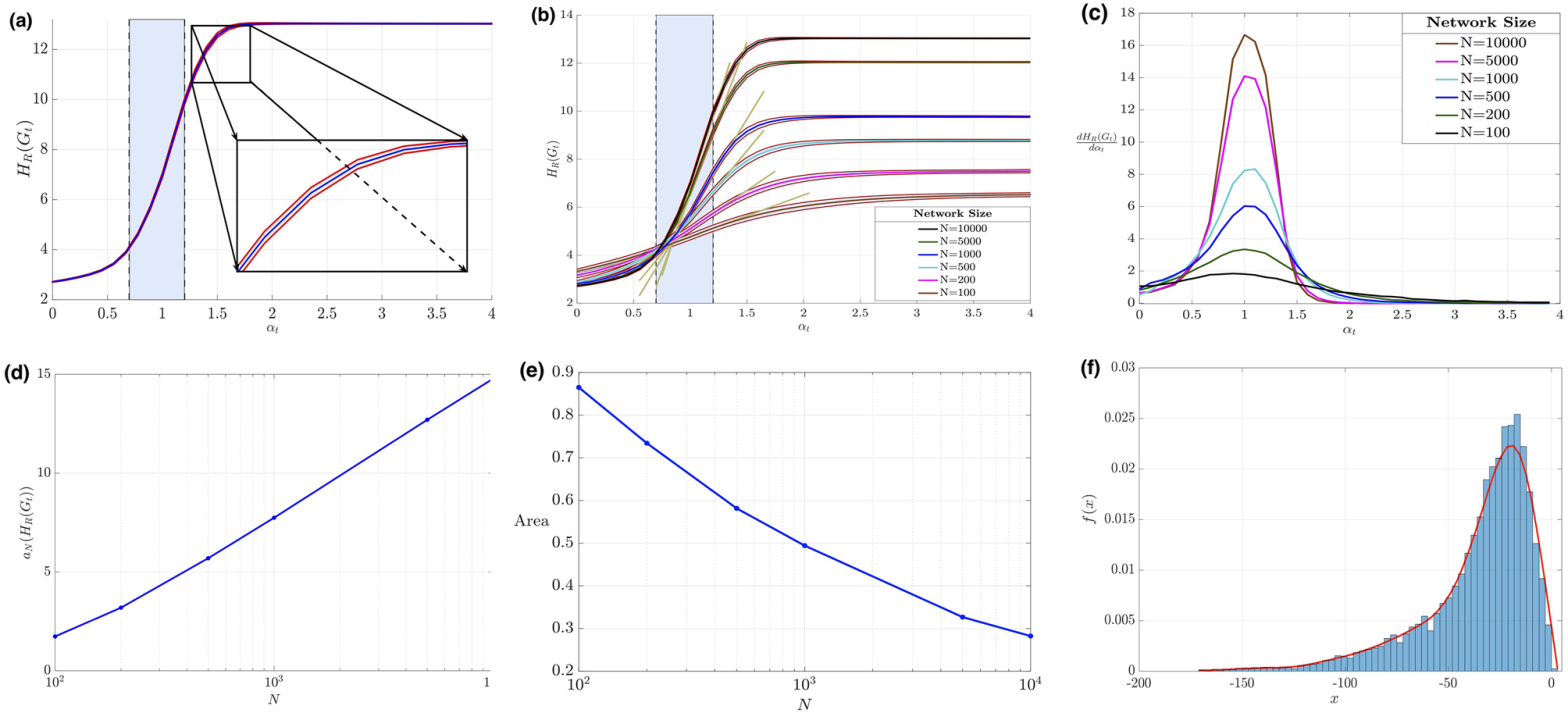
(**a**) Forman–Ricci network entropy of random growing network of size $N=10{,}000$ performed at $10^3$ realizations. The blue curve is the expected value of the Forman–Ricci network entropy, and the two red curves define the 95% mean confidence interval. (**b**) Forman–Ricci network entropy and (**c**) its first derivative for random growing network of sizes $N=100, 200, 500, 1000, 5000$ and 10, 000 where the first derivative is used as a tool to detect the phase transition of a random growing network. (**d**) The slope of the Forman–Ricci network entropy’s rise versus the network size *N*. (**e**) Shaded area vs. the network size *N*. (**f**) Forman–Ricci curvature distribution associated to a random growing weighted network with parameters, size of the network $N = 1000$ and attachment probability parameter $\alpha = 0.5$.

Figure 2a, illustrates the Forman–Ricci network entropy for a network of $N=10{,}000$ nodes, as a function of the parameter $\alpha _t$ (the blue curve). The blue curve is the result of a Monte-Carlo simulation performed for $10^3$ realizations of the random growing network. The two red curves define $95\%$ mean confidence interval (shaded region in the zoomed part). From Fig. 2a, we can observe that the network has two distinct states and a transition period (cyan shaded region). The first state with a “quasi-constant” Forman-RC network entropy occurs for $\alpha _t \le 0.6$, and a second stable state occurring for $\alpha _t \ge 1.45$ achieving constant entropy value. From the figure we can observe the transition between the two states happening around $0.6\le \alpha _t\le 1.45$; however, providing an accurate estimation of the boundary of the phases is not trivial. In the “Methods” section, we provide an approach to quantify the lower and upper boundaries that separates the different phases by observing the first-order derivative of the Forman-RC network entropy (Fig. 2c). Interestingly, the significant change is happening around $\alpha _t=1$ (the cyan shaded area) which corresponds to the classical Barabási-Albert model. This highlights the change of the topology of such network for $\alpha _t<1$, $\alpha _t=1$ and $\alpha _t>1$. Hence, we can conclude that the random network, under study, experiences a phase transition which separates two major states of the network. This result is consistent with the claim of reference^7^ where the authors showed that different behaviors arise for the network considering different values of $\alpha _t$. Therefore, $\alpha _t$ is a crucial parameter that defines the topology of the network.

Figure 2b summarizes similar experiments obtained for different network sizes ($N=100$, 200, 500, 1000 and 5000 nodes). From the aforementioned figure, we can see that the Forman–Ricci network entropy has the same shape analogous to a sigmoid curve. We can remark that the midpoint is fixed; the cyan bar is consistent around $\alpha _t=1$. Observing the first-order derivative of the Forman-RC network entropy versus the time-dependent parameter $\alpha _t$ as in Fig. 2c, we can see, for each fixed network size *N*, the first derivative of the Forman-RC network entropy is a unimodal curve where the “energy” is concentrated around the same value $\alpha _t = 1$. This defines the peak of the first derivative of the entropic measure which confirms the transition is occurring exactly at $\alpha _t=1$. The other observation from the distinct plots is that the Forman-RC network entropy, being in the first state ($\alpha _t \le 0.6$), does not vary too much while changing the size of network *N*, but it increases for large network sizes *N* when the network is in the second state ($\alpha _t \ge 1.45$). Furthermore, we observe that the shaded region (zoomed part mentioned before) shrinks as *N* increases (see Fig. 2e); this result is intuitive since we have more samples of the Forman-RC in large networks. Hence, we will have an accurate estimator of the probability density function corresponding to the Forman-RC in the network. Thus, the error relative to the wavelet-based estimator will decrease which will narrow the $95\%$ mean confidence interval. As we can see from the tangent lines (marked in dark khaki color) corresponding to each plot in the transition phase ($\alpha _t=1$), the slope of the rise of the Forman–Ricci network entropy is changing while growing the size of the network *N*. Figure 2d shows the relationship between the slope of rise of the Forman–Ricci network entropy $H_R(G_t)$ denoted $a_N(H_R(G_t))$ and the network size *N*. It is clear that the slope and the size *N* are positively correlated which aligns with our intuition (for infinitely large network sizes, the Forman–Ricci network entropy plot will be similar to a step function). Additionally, we perform linear regression analysis and deduce that for large value of *N*, we have $a_N \log N^\delta$ where $\delta = 2.9 \pm 0.1$. An interesting observation from this figure is that the slope varies linearly versus $\log N$. More investigation will be done in the future to explain this relationship. Finally in Fig. 2f, we show an example of the histogram (blue rectangles) of the Forman-RC distribution associated with the random growing network for $N = 1000$ and $\alpha = 0.5$ as well as the estimated distribution *f*(*x*) using the wavelet-based estimator (red curve) versus the curvature of edges denoted *x*.

### Small-world weighted network

**Figure 3:**
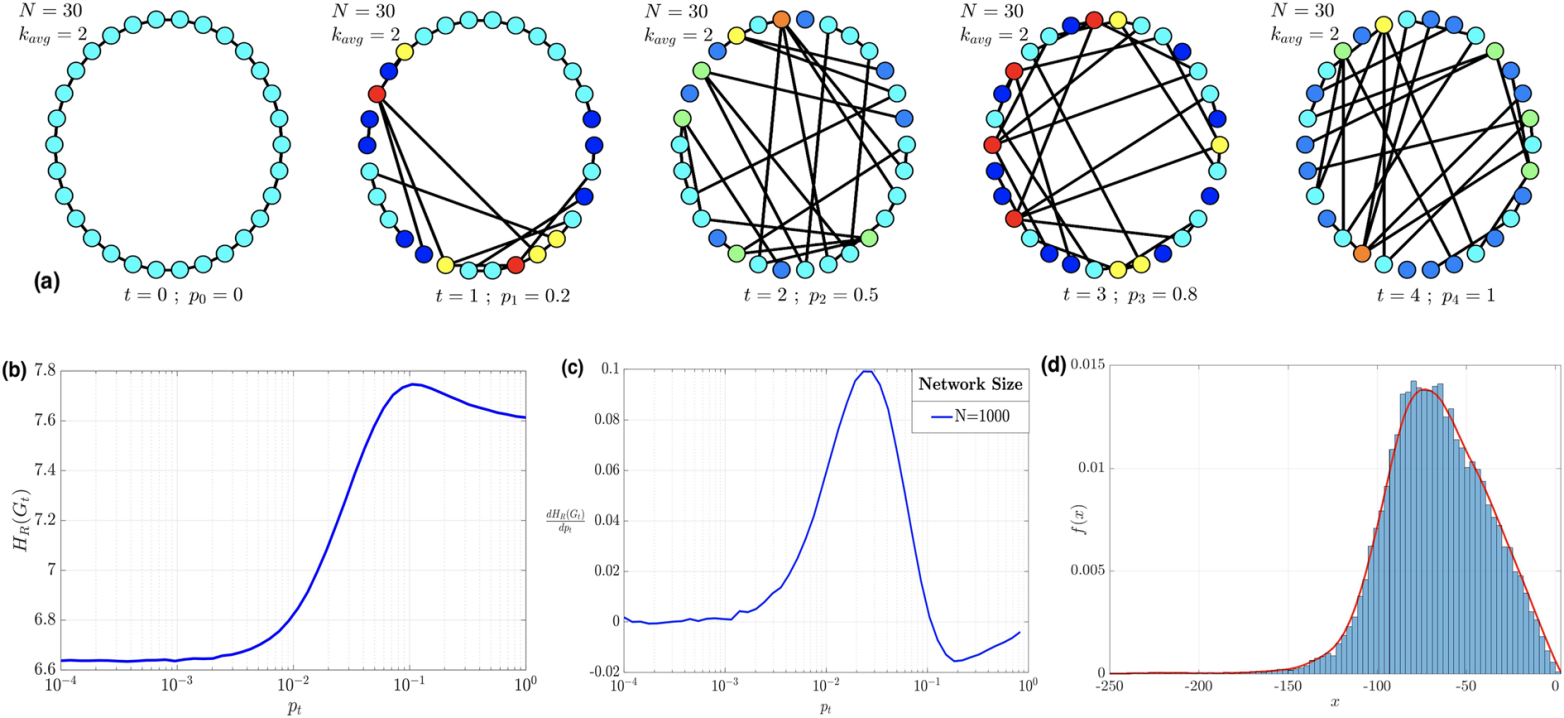
(**a**) Time evolution of a small-world network from regular to random with varying rewiring probability $p_t$ changing in time. Each time *t* corresponds to a value of $p_t$ used to generate the network $G_t$ containing $N=30$ nodes having an average degree $k_{avg}=2$. (**b**) Forman–Ricci network entropy, (**c**) first derivative of the Forman–Ricci network entropy, and (**d**) Forman–Ricci curvature distribution associated to small-world networks generated according Watts–Strogatz model (i.e., rewired graphs with rewiring probability parameter *p*), $N=1000$, $K=50$.

To validate the proposed approach, we design a second scenario where we consider the case of small-world networks generated according to the Watts–Strogatz model that depends on three parameters (*N*: number of nodes, *p*: rewiring probability and *K*: average degree). Here, we assume that at each time instant we generate a weighted network with rewiring probability *p* between 0 (Regular network) and 1 (Random network), and as the time evolves the parameter *p* increases. Our goal is to study the structure of the network and detect when it changes. To illustrate this phenomenon, Fig. 3a shows five network snapshots wherein the rewiring probability $p_t$ takes the values $p_t = \{0, 0.2, 0.5, 0.8, 1\}$ at times $t = \{0, 1, 2, 3, 4\}$, respectively. The network is evolving from a regular network (i.e., $p_0 = 0$), where each node is only connected to its neighbors, to a random network (i.e., $p_4 = 1$). Hence, the topological properties of this network are changing while the rewiring probability is changing in time. In Fig. 3b, we consider a random network of size $N=1000$ nodes, we plot the Forman–Ricci network entropy versus the rewiring probability parameter *p*. The blue curve is determined by running a Monte–Carlo simulation performed for $10^3$ realizations of the Watts–Strogatz network. From Fig. 3b, we can observe that the network has two distinct states (State 1: $p \le 10^{-2}$, State 2: $p \ge 10^{-1}$). For the values $10^{-2} \le p \le 10^{-1}$, we can see a phase transition between the two states. Similar to the analysis done for the random growing networks case, Fig. 3c shows the first derivative of the Forman-RC network entropy. More specifically, the transition is happening between $p=0.02$ and $p=0.03$. This result matches perfectly with the observation presented in^6^, where they also observe the properties of a small-world network for a similar range of rewiring probability. However, their analysis was based on evaluating the clustering coefficient and the average path length (the transition regime is characterized by a high clustering coefficient and short path length). Similarly, in Fig. 3d, we plot the estimated Forman–Ricci curvature distribution and the histogram for a small-world network of size $N = 1000$ with average degree $K=50$ and a rewiring probability $p = 0.5$. In this case, the support is wider than the one corresponding to the Forman–Ricci curvature distribution for a random growing network. In both cases, by comparing both histograms and their estimated distributions, we can see that the density estimators perform well. This result is expected since we have a relatively large network size $N = 1000$, thus a large sample size for density estimation.

### Stochastic Block model with planted community structure

**Figure 4:**
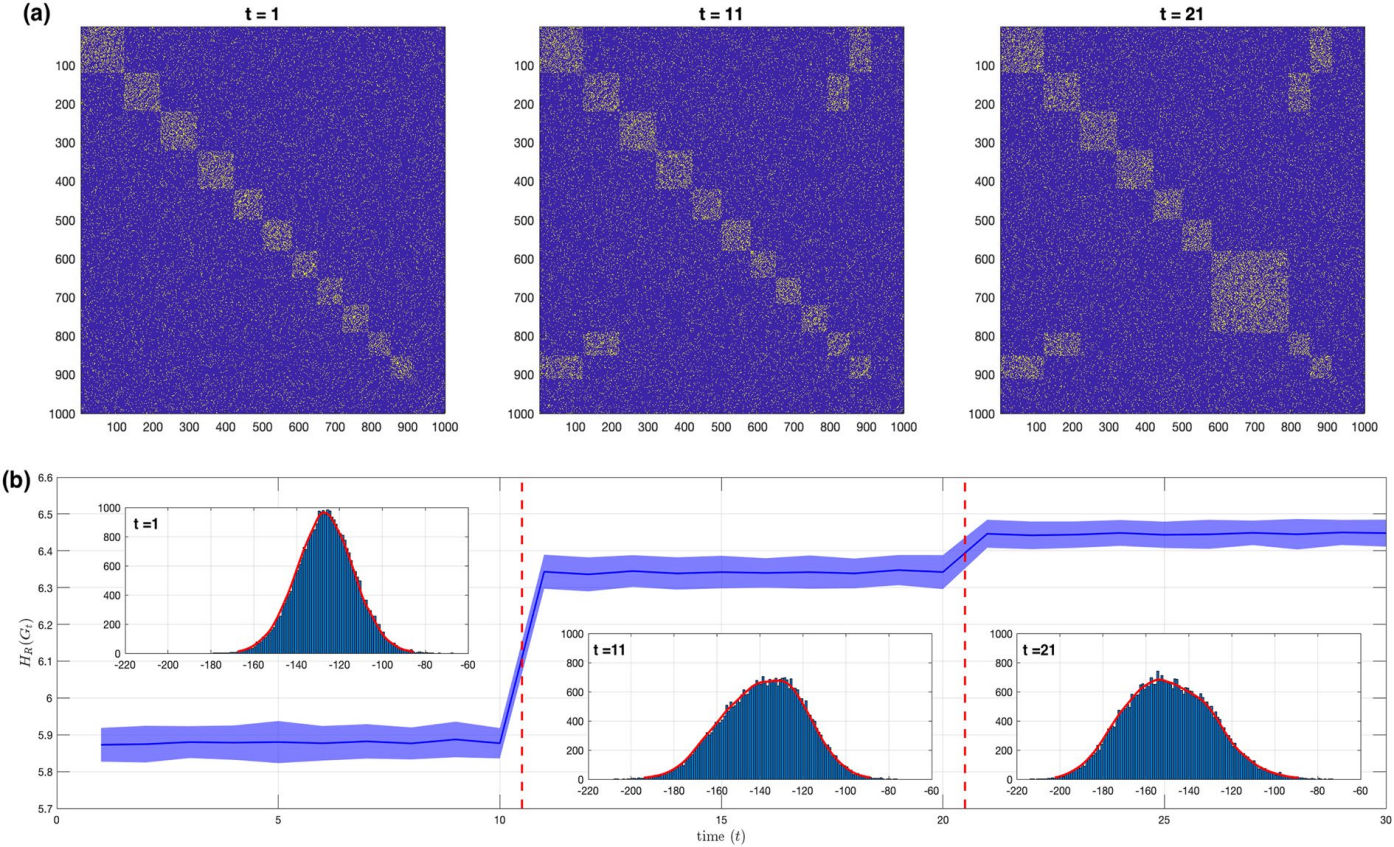
Forman–Ricci network entropy analysis of time-varying stochastic block models (SBM) for $T=30$ network snapshots. Phase transitions occur at $t=11$ and $t=21$ by merging of communities. The stable time periods are characterized by fixed community structures occurring at the following time intervals $t = \{[1,10],[11,20],[21,30]\}$. (**a**) Heatmap images of select adjacency matrices at different stable time periods. Time instants $t=1,11$,and 21 are shown. Densely packed yellow pixels (edges) indicate community structures. (**b**) Forman–Ricci network entropy over the entire time period up to time $t=T$. Blue line and blue shaded region indicate the sample mean and sample standard deviation bands for 100 realizations, respectively. Red dashed lines indicate the phase transition points. Inscribed plots within the main plot show the Forman–Ricci curvature histograms and density estimates for one realization at time instants $t=1,11$, and 21, respectively.

We investigate the network phase transition phenomena as we observe changes in the core community structures over time. We follow the time-varying complex network setup similar to^8^ where we utilize the Stochastic Block model (SBM) as the network generating model for $N=1000$ nodes and $T=30$ network snapshots. At $t=1$, 11 densely connected communities are generated and labelled from $0,\ldots ,10$ of size 120, 100, 100, 100, 80, 80, 70, 70, 70, 60, and 60, respectively, with the remaining 90 nodes not forming any community. Abrupt changes to the community structure is induced at (i) $t=11$ with community 10 merged to 0 and 9 merged to 1, and at (ii) $t=21$ with communities 7 and 8 merged to 6. Figure 4a shows the heatmap images of the adjacency matrix for the following time stamps $t=1,11$, and 21, respectively. Yellow pixels denote edges. Squares of densely packed yellow pixels denote community structures. The community structure is represented as a low-ranked matrix *L*(*t*) which is held invariant between phase transition points. The density of connections $p_c = 0.2$ within community is generated as follows: $L_{in} \overset{\text {i.i.d.}}{\sim } \text {Bernoulli}(p_c)$ and $L_{out} = 0$, where $L_{in}$ denotes connections within communities and $L_{out}$ otherwise. Sparse noise *S*(*t*) represents the perturbations and are allowed to vary freely at each time instant. The noise density is set at $p_s=0.1$. *S*(*t*) is generated as follows: $P(S_{ij}=1)=0.05$, $P(S_{ij}=-1)=0.05$, and $P(S_{ij}=0)=0.9$. Finally, the sum of *L* and *S* is projected as an adjacency matrix: $A(t) = {\mathscr {P}}_\Omega [L(t) + S(t)]$, thus keeping binary values and symmetry.

In this scenario, we investigate the curvature analysis on detecting phase transitions that are characterized by changes to the core community structure with the presence of noise. Figure 4b shows the Forman–Ricci network entropy over the entire time period up to $t=T$. The blue solid line represents the sample means, and the blue shaded region represents the sample standard deviation bands for 100 realizations. The red dashed lines indicate the phase transitions. The inscribed plots within the main figure show the histograms and density estimates of the Forman–Ricci curvatures for the selected time instants $t=1,11$, and 21, respectively. From Fig. 4b, we can observe that the mean Forman–Ricci network entropy has mean values at 5.88, 6.34 and 6.45 and standard deviation values at 0.0044, 0.0024 and 0.0025 for each of the three stable time periods, respectively, wherein the community structures are invariant. Variations in the network entropy within the stable periods are due to the induced perturbations. For this setup, despite the induced perturbations, the Forman–Ricci network entropy can deduce the phase transition points. Pre-processing the data by filtering out the noise is expected to further improve the results. This example shows that Forman–Ricci curvature analysis method is able to detect phase transitions due to changes in core community structure. This method does not need to perform a direct community detection method at each time instant which can lead to very high computation complexity.

### Weighted multifractal networks (WMFNs)

**Figure 5:**
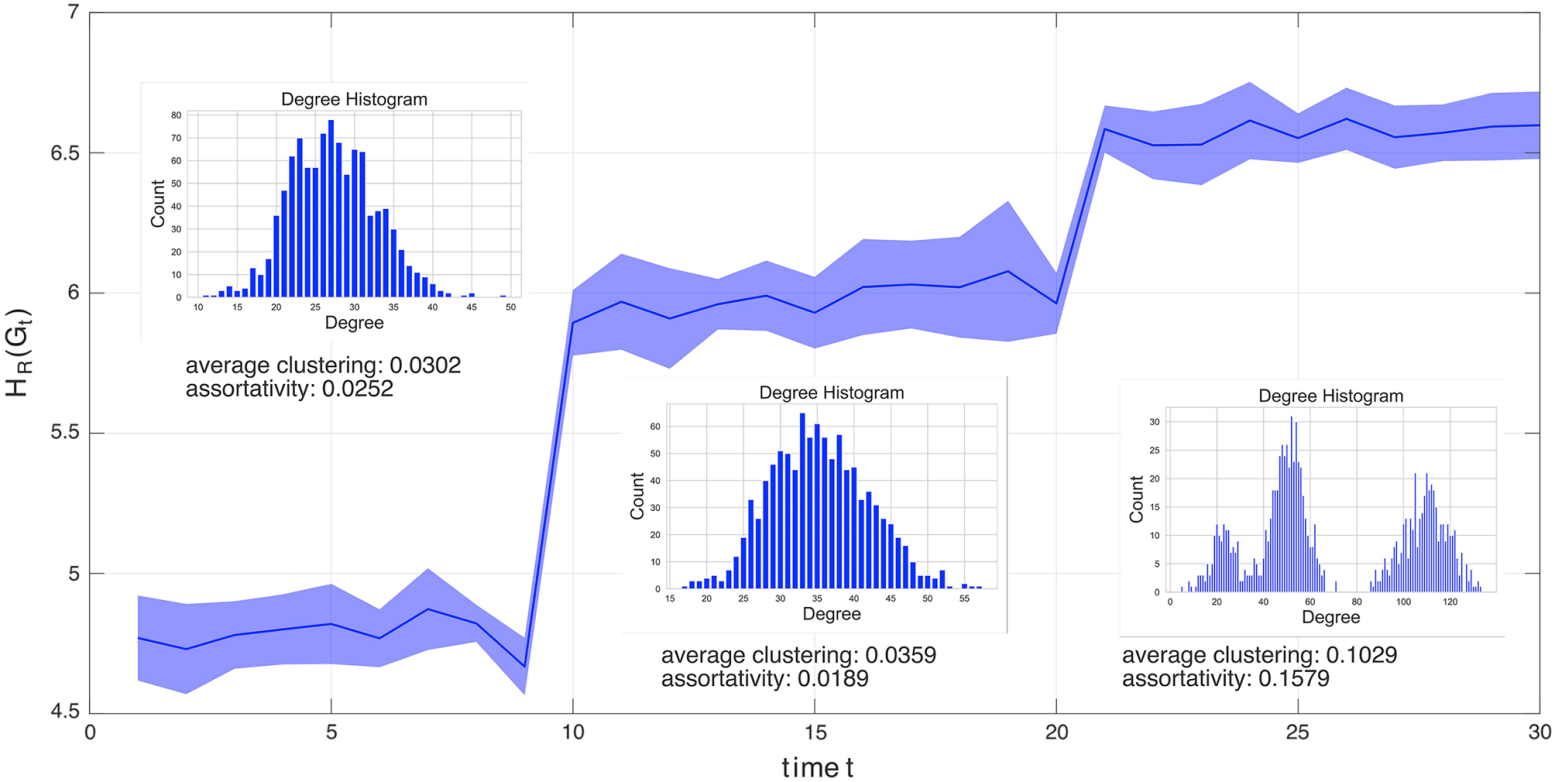
Forman–Ricci network entropy analysis of time-varying weighted multifractal network models^21,30^ for $T = 30$ network snapshots for $N=1000$ network size. Phase transitions occur at $t = 10$ and $t = 20$ by changes to the linkage probabilities *p*. The “stable” time periods are characterized by the same weighted multifractal network generator parameters with small perturbations across time occurring at the following time intervals [1, 9], [10, 19] and [20, 30]. A sample degree histogram is provided for each of the “stable” regions, as well as the average clustering and associativity. The simulation is repeated for 10 iterations.

Similar to the Stochastic Block Model with Planted Community Structure case study (Fig. 4), the proposed Forman–Ricci network entropy metric is capable of detecting multiple phase transitions in WMFNs^21^. In contrast to the SBM example where changes to the community structures cause the phase transition, the changes in the generating parameters drive the WMFN phase transition. The result of the case study on WMFNs is presented in Fig. 5. Here, each network snapshot has $N=1000$ nodes. The multifractal generating model is recursively generated from a self-similar division of a simple square structure. We use $R=2$ layer unit-squares, $M=2$ intervals and $K=3$ self-similar operations. The linkage probabilities $p_i = [p_i(1), p_i(2)]$ has two sets of matrix values due to the chosen $R=2$ number of layers. We keep the interval lengths fixed for all times with value $L = [0.75, 0.25]$. At the generating parameter change points $t=\{10,20\}$, the proposed method detects the phase transition through a remarkable change in the Forman–Ricci network entropy value. In fact, the proposed metric is capable of quantifying a state of a WMFN that is uniquely defined by the generating parameters which characterizes the topology of the network.

As shown in Fig. 5, we can observe that we have three different phases which is compatible with the values of the chosen generator parameters. The mean Forman–Ricci network entropy is marked with a solid blue line. The fluctuations in entropy across time *t* within the each of the stable parameter is due to the induced perturbation in the generating parameters. The Forman–Ricci network entropy is calculated using a Monte-Carlo simulation performed for multiple realizations of the WMFN. The shaded blue region defines a $95\%$ confidence interval for the mean value of the Forman–Ricci network entropy. At $t=10$ and $t=20$, the generating parameter transitions from $p_1$ to $p_2$ and $p_2$ to $p_3$, respectively, resulting in a significant change in network structure as described by the degree distribution and average clustering coefficients. The KL divergence for the degree distributions at the phase transitions are $\textrm{KLdiv}(1\rightarrow 2) = 0.7446$ and $\textrm{KLdiv}(2\rightarrow 3) = 1.2967$. The change in average clustering coefficients are $\Delta |C(1\rightarrow 2)| = 0.006$ and $\Delta |C(2\rightarrow 3)| = 0.067$. This corresponds to a Forman–Ricci network entropy change $|\Delta H_R (1 \rightarrow 2)| =|H_R(G_{10})- H_R(G_{9})| \approx 2.2$ and $|\Delta H_R (2 \rightarrow 3)| =|H_R(G_{20})- H_R(G_{19})| \approx 0.7$. Only a subtle change in network structure occurs as described by the difference in degree distributions ($\textrm{KLdiv}(1\rightarrow 2)$) and average clustering coefficients ($\Delta |C(1\rightarrow 2)|$) when comparing the parameter change between $p_1$ and $p_2$ (time intervals 1 and 2). Nevertheless, this results in a detectable phase transition with a Forman–Ricci network entropy change of $|\Delta H_R(1 \rightarrow 2)|$. We can utilize the first derivative of the Forman–Ricci network entropy as a tool to quantitatively detect the phase transition region (see “Methods” section on Transition boundary quantification, Eq. (5)).

**Figure 6:**
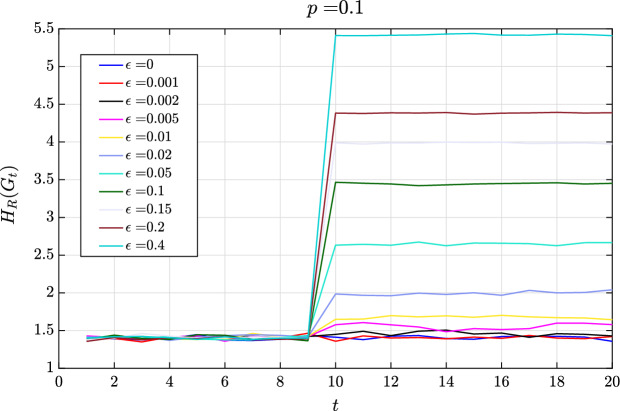
Forman–Ricci network entropy with respect to perturbations in the linkage probability for a monofractal network.

We further analyze the impact of perturbation at different magnitudes to the linkage probabilities for a monofractal network. For a monofractal network, the linkage probabilities are all uniformly set for all box-coverings. For this example, we start at a linkage probability of $p = 0.1\cdot \mathbbm {1}_{2}$, where $\mathbbm {1}_{n}$ is an *n*-by-*n* matrix of ones. At $t=10$, the linkage probability is perturbed by $\epsilon$, $p = (0.1 + \epsilon )\cdot \mathbbm {1}_{2}$. From Fig. 6, perturbations greater than $1\%$ of the initial *p* value results in detectable phase transition. Meanwhile, perturbations smaller than $1\%$ of the initial *p* value results in very small changes in network entropy for a successful phase transition detection.

### Artificial neural networks

Due to the diverse range of applications attributed to deep neural networks (DNNs), it is imperative to comprehend their intricacies in order to enhance the design of optimal architectures that are capable of effectively addressing various real-world machine learning problems. Analyzing the non-trivial interactions between the nodes of a DNN architecture is a key step towards elucidating the underlying mechanism governing the network throughout the training process. In this vein, our aim is to develop an approach that unveils the interplay between the geometric properties of the artificial neural network and the corresponding learning task.

**Figure 7:**
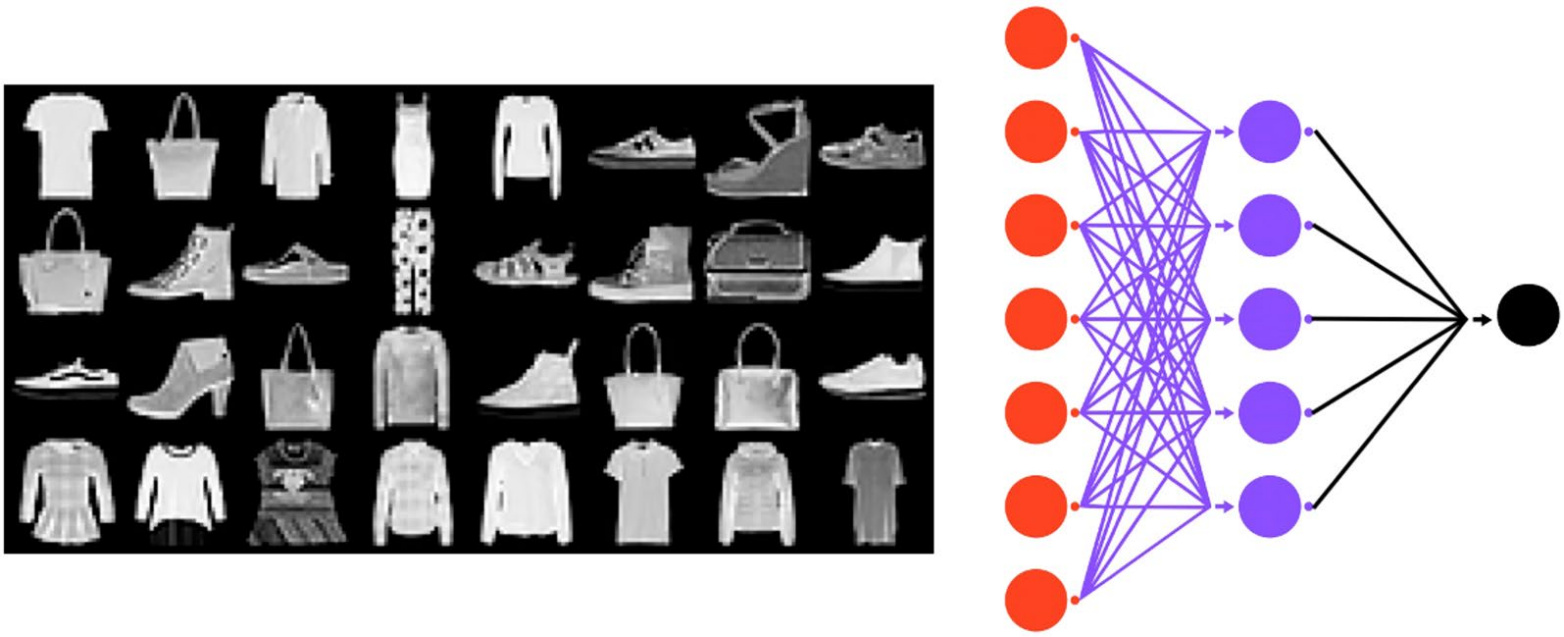
Phase transition analysis for the DNN architecture training using the Fashion MNIST dataset. Data samples are $28 \times 28$ pixel images from the 10 classes of the Fashion-MNIST dataset using a NN architecture of 784-32-10 input-hidden-output neurons.

In pursuit of this endeavor, we present a case study wherein we investigate the hypothesis that our proposed framework has the capacity to detect topological phase transitions during the training process of a neural network architecture. Such transitions can offer valuable insights into the state of the DNN. Specifically, we conduct the experiment of training a NN architecture with dimensions 784-32-10 on the Fashion-MNIST dataset^31^ (Fig. 7). It is worth noting that the first layer of the network is specifically designed to reshape the input grayscale images, which have a size of $28 \times 28$ pixels.

**Figure 8:**
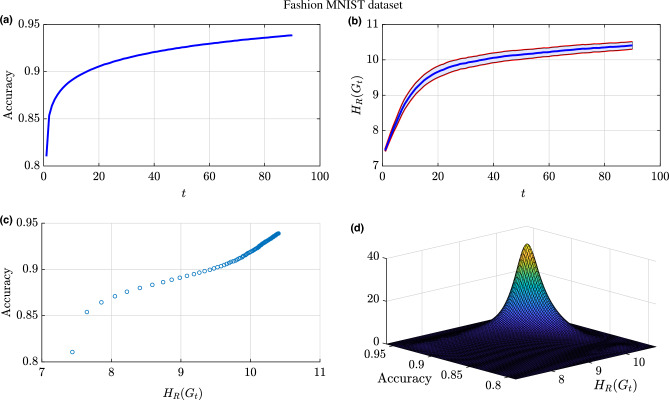
Results for training NN architecture (784-32-10) for the purpose of classifying the Fashion MNIST dataset: (**a**) Training accuracy over the number of training epochs. (**b**) Forman–Ricci network entropy over the number of training epochs. The shaded region represents a $95\%$ confidence interval for the mean Forman–Ricci network entropy calculated over multiple experiments. (**c**) Accuracy versus the Forman–Ricci network entropy. (**d**) Joint distribution of the accuracy values and the Forman–Ricci network entropy.

We train the DNN on the Fashion-MNIST dataset for 90 epochs and record the accuracy and the Forman–Ricci network entropy at each training epoch. At around epoch 30, the classification accuracy starts to plateau around 0.915 which indicates that the additional training samples are not significantly helping the learning process (Fig. 8a). Figure 8b shows the Forman–Ricci network entropy over the entire training epochs with the shaded region indicating a $95\%$ confidence interval for the mean Forman–Ricci network entropy calculated over multiple experiments. Similar to the accuracy plot, the Forman–Ricci network entropy is also an increasing function versus the training epochs. As we can observe, the Forman–Ricci network entropy plot has a similar behavior as the accuracy plot. As shown in Fig. 8c, the scatter plot shows a strong, positive correlation between accuracy and Forman–Ricci network entropy during the training process. During the training process of a NN architecture, the accuracy starts increasing (and the loss functions starts decreasing) versus the training epochs until convergence (i.e., minimal additional improvement from additional training samples). Consequently, there are two phases during training a NN architecture over multiple epochs. From the positive correlation presented in Fig. 8c and the information provided in Fig.8b, we can conclude that the Forman–Ricci entropy metric is capable of detecting the learning phase change in during DNN training.

In order to deeply analyze the connection between the accuracy and the Forman–Ricci network entropy, we have estimated the joint probability distribution function shown in Fig. 8d with a concentration in probability around accuracy values between 0.915 and 0.94 and entropy values between 9.9 and 10.4. This indicates a state of learning saturation as indicated with a slow rate of change in accuracy and Forman–Ricci network entropy convergence. This relates the geometry of the network of neurons in a NN architecture with the learning process. In the line with this analysis, we have computed the Pearson correlation coefficient between the Forman–Ricci network entropy and the accuracy and we get $\rho =0.9735$. The observed findings demonstrate a strong positive correlation between the aforementioned metrics, providing robust evidence that substantiates our initial hypothesis. This compelling outcome confirms the efficacy of the proposed framework in accurately detecting and quantifying topological changes that occur throughout the training process of a neural network architecture.

**Figure 9:**
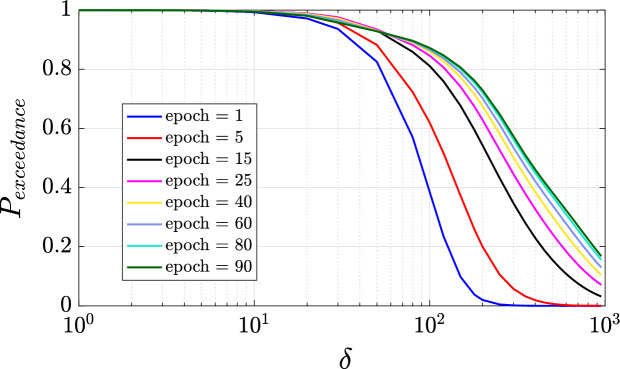
Forman–Ricci curvature exceedance probability as a function of threshold $\delta$.

Next, we analyze the exceedance probability of the Forman–Ricci curvature values associated to the edges present in network at each epoch. We have remarked that all the Forman–Ricci curvature values are negative, so we considered the magnitude of the Forman–Ricci curvature in the exceedance probability evaluation, $P_{exceedance} = P(|Ric_F| > \delta )$. The Forman–Ricci curvature exceedance probability plot is shown in Fig. 9. As the NN architecture is trained from the initial setting up to epoch 15, we see an increase in the Forman–Ricci curvature exceedance probability for threshold values $\delta < 55$. The opposite happens for the same threshold values $\delta < 55$ with the Forman–Ricci curvature exceedance probability decreasing as the training continues from epoch 25–90. We note that the exceedance probability curves cross around $\delta \approx 55$ with the first crossing occurring between epochs 15 and 25. From Fig. 8a,b, we also observe a significant decrease in the rate of change in both accuracy and the Forman–Ricci network entropy between epochs 15 and 25. This behavior can be attributed to changes in network topology with a critical phase change occurring between these training epochs.

To further validate the efficacy of our framework and the insights garnered from the previous neural network experiment, we conducted additional experiments employing a distinct dataset, namely CIFAR-10. The aim was to fortify the credibility and generalizability of our proposed framework. Specifically, we have replicated the previous experiment by employing the same architecture (1024-32-10) utilized for classifying the Fashion-MNIST dataset.

It is noteworthy to highlight that we have tested our framework on grayscale images, which necessitated the conversion of the RGB images from the CIFAR-10 dataset into grayscale representations. Subsequently, the grayscale images, characterized by a single channel instead of the original three channels in RGB images, were utilized as input to the neural network architecture. Consequently, the first layer of the network was designed to accommodate the transformed grayscale image inputs. It consisted of 1024 nodes, aligning with the dimensions of the grayscale images, which have a size of $32 \times 32$ pixels.

**Figure 10:**
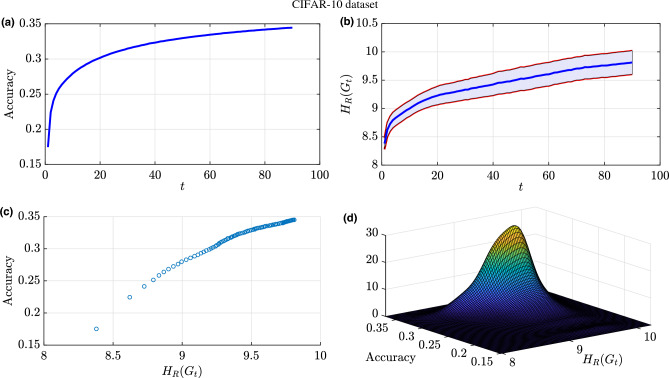
Results for training NN architecture (1024-32-10) for the purpose of classifying the CIFAR-10 dataset: (**a**) Training accuracy over the number of training epochs. (**b**) Forman–Ricci network entropy over the number of training epochs. The shaded region represents a $95\%$ confidence interval for the mean Forman–Ricci network entropy calculated over multiple experiments. (**c**) Accuracy versus the Forman–Ricci network entropy. (**d**) Joint distribution of the accuracy values and the Forman–Ricci network entropy.

Due to the conversion of the images and the utilization of a simplistic architecture for the learning task, the achieved accuracy is not high enough (i.e., 0.35) as shown in Fig. 10a. Similarly to the results illustrated in Fig. 8, we observe that the Forman–Ricci network entropy is a monotonically increasing function throughout the training process. However, we can observe that the rate of change of the Forman–Ricci network entropy diminishes as the number of epochs increases (Fig. 10b), which corresponds to a similar behavior observed in the accuracy metric. Both metrics exhibit comparable trends, thereby corroborating our earlier observation concerning the intrinsic relationship between the the Forman–Ricci network entropy and the training accuracy as shown in Fig. 10c,d. This experiment is designed to confirm the correlation between the accuracy and the Forman–Ricci network entropy. Consistent with the previous experiment, we performed a calculation of the Pearson correlation coefficient between the Forman–Ricci network entropy and the accuracy and we get $\rho =0.9809$.

**Figure 11:**
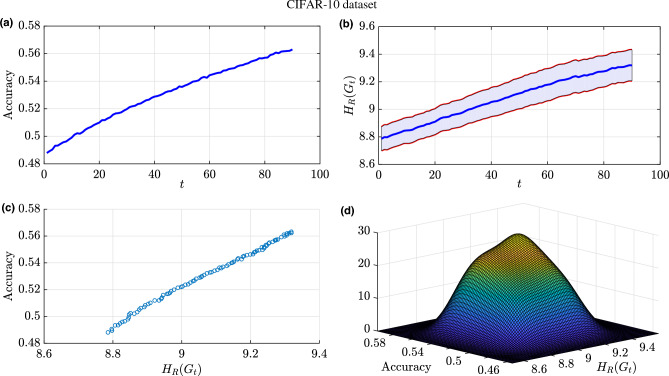
Results for training NN architecture (1024-256-64-32-10) for the purpose of classifying the CIFAR-10 dataset: (**a**) Training accuracy over the number of training epochs. (**b**) Forman–Ricci network entropy over the number of training epochs. The shaded region represents a $95\%$ confidence interval for the mean Forman–Ricci network entropy calculated over multiple experiments. (**c**) Accuracy versus the Forman–Ricci network entropy. (**d**) Joint distribution of the accuracy values and the Forman–Ricci network entropy.

In order to establish and confirm the robust correlation between the Forman–Ricci network entropy and the training accuracy of a NN architecture, we have designed an experiment where we have changed the NN architecture to classify the grayscale images from the CIFAR-10 dataset. The classification task was performed using a deeper and more complex NN architecture with dimensions 1024-256-64-32-10, employing the rectified linear unit (ReLU) as activation functions. This architectural modification allowed for an increase in accuracy, reaching a value of 0.563, as demonstrated in Fig. 11a.

Furthermore, in line with our previous observations, we can notice that the Forman–Ricci network entropy is consistently exhibited an upward trend throughout the training phase, as depicted in Fig. 11b and this affirms that running multi-layer perceptron tends to increase the Forman–Ricci network entropy during the training phase. Notably, in this experiment, the Pearson correlation coefficient between the Forman–Ricci network entropy and the accuracy is equal to $\rho =0.996$, further emphasizing the strong correlation between both metrics which is also depicted in Fig. 11c,d. We hypothesize that the correlation strength intensifies as the architecture’s depth increases. With this analysis, we have built a connection between the topological properties of the nodes in a NN architecture and its performance. On one hand, the Forman–Ricci network entropy is strongly correlated to the accuracy throughout the training process. On the other hand, the proposed metric is capable of detecting phase transition in NN topology during the training process. In fact, our experiments yielded promising results, indicating that analyzing the training process using curvature via the Forman–Ricci network entropy is relevant and useful for gaining insights into the performance of neural networks. Therefore, the provided network geometry analysis can be a key step towards understanding learning in DNNs. One potential application area is the Forman–Ricci network entropy can help find the number of epochs needed to train a DNN (i.e., detect when the accuracy/loss functions converges). Hence, this entropic metric that we have presented can be a new useful tool to avoid overfitting. Also, the dropout technique used in DNN to prevent overfitting can now be performed by ignoring the nodes having a constant Forman–Ricci curvature throughout the training process and not contributing to the Forman–Ricci network entropy.

**Figure 12:**
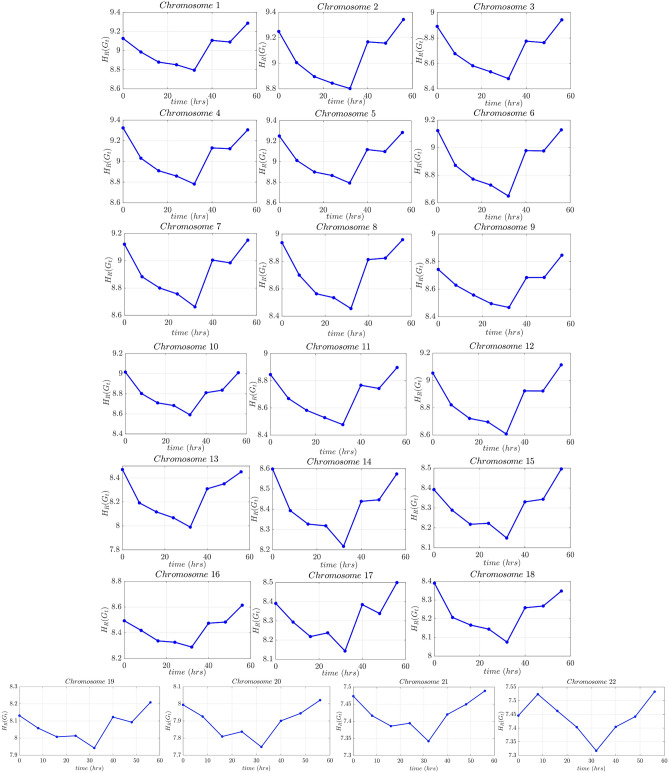
Forman–Ricci network entropy of time-varying Hi-C matrices collected from cell cycle- and circadian rhythm-synchronized proliferating human fibroblasts of normal karyotype (for each chromosomes 1–22) collected every 8 h over a 56hr time period. Minimum Forman-RC network entropy matches the bifurcation point at $t=32$ h in the cell state trajectory for fibroblasts being reprogrammed into the myogenic lineage.

### Real-world dataset: genome-wide chromosome conformation capture (Hi-C)

Besides the artificially generated networks, we tested this algorithm on a real-world dataset from biology. For this, we analyzed time series corresponding to a genome-wide chromosome conformation capture (Hi-C) matrix dataset. Hi-C matrices encode how often two genomic loci contact each other in 3D space at a given time. These data can be constructed into a contact matrix *A* for each chromosome, where the number of contacts between locus *i* and locus *j* resides in element $A_{ij}$. For this analysis, Hi-C matrices generated from cell cycle and circadian rhythm synchronized human fibroblasts were obtained from^32^. The samples of the Hi-C matrices are collected every 8 h during the period of 56 h; more details about this dataset can be found in^32,33^. These Hi-C matrices are interpreted as a representation of a time varying complex network, where the nodes are the genomic loci, and the entries of the Hi-C matrix correspond to edges of the network which indicate the contact between genomic loci. The goal is to use our framework to analyze these networks, hence, discovering important features in Hi-C matrix data. Based on the links and their strength, the Forman-RC helps to identify local properties of the network such as the bridge nodes in a network and to distinguish between different regions. In the context of Hi-C matrices, the regions can be seen as active and inactive transcriptional chromatin domains, and the bridge nodes can be interpreted as architectural buffers between the regions.

Figure 12 demonstrates that the proposed Forman-RC inspired phase transition detection method can identify a change in the curvature established between chromosomes during fibroblast proliferation. As we can see from the Forman–Ricci network entropy plots, we have two regimes: during the first five time points from $t=0~$ h to $t=32$ h, the network tends to minimize the Forman–Ricci network entropy. From a biology perspective, during this period the cells are completing their first synchronized cell cycle. In contrast, during the time interval between $t=32$ h and $t=40$ h, the cells have completed their first cell cycle and start to become un-synchronized during the remaining time points where the network tends to increase the Forman–Ricci network entropy. Interestingly, this result matches perfectly the biological experimentation and observations in^33^, where the authors demonstrated that a bifurcation point occurs at $t=32$ h in the cell state trajectory for fibroblasts being reprogrammed into the myogenic lineage. Consequently, the proposed framework allows us to detect architectural reorganization and changes in the genome architectural dynamics.

### Real-world dataset: roll call data of the US senate

Time-varying CNs can also model the interactions, affinities, similarities or relationships between nodes (politicians) in a social (political) network in order to predict unobserved trends (tendencies) in the political life. In this context, we map the voting records of the US Senate from 1979 (96-th Congress) to 2012 (112-th Congress)) to time-varying CNs. The networks are constructed in a similar way as in^3^. More precisely, the idea is to take the records of the 100 Senate members, from the website voteview, then the *yes* and *no* decisions are mapped to 1 and 0, respectively. It is obvious that the Senate members cannot be all continuously re-elected during the entire period (1979–2012); also some of them retired. This has an impact on the number of nodes within the networks. To solve this issue, Roy, et al. proposed in^3^ a continuous record of votes, by looking at the voting pattern of each Senate seat. Among the total number of 12, 129 votes during the period we examined, only 7949 votes are considered to construct the dataset, the remaining votes exhibits a conformity more than $75\%$ in the *yes*/*no* direction. Note also that the data has some missing values, due to some absentees. The authors of the dataset insert a value of 1 or 0 corresponding to the member’s party decision obtained by majority polling on that particular vote (more details are provided in^3^). It is worth mentioning that these time varying graphs are binary (unweighted). We believe that by analyzing these matrices individually, we are capturing the time evolving voting dependency structure between Senate seats. For this reason, we are analyzing a consecutive block of matrices together by looking at the average matrix of different votes. The intuition behind this is to compute the probability of having an agreement between any two nodes. Here, we combine each 240 votes into a single matrix that measures the probability of agreement. The size 240 is the average number of votes per year during 33 years. Hence, we have 33 time points (networks).

**Figure 13:**
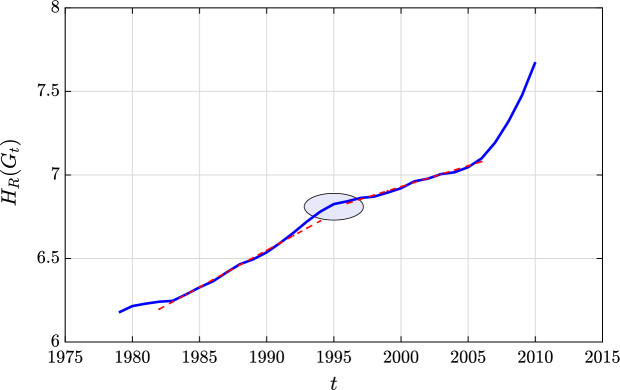
Forman–Ricci network entropy of the combined US senate data from 1979–2012. Red dashed-lines are the linear regression fit lines for the periods 1986–1994 and 2000–2012. The circled region indicates the end of the Conservative Coalition starting around 1994.

Figure 13 illustrates the evolution of the Forman–Ricci network entropy of the constructed time-varying complex networks that encode the voting records of the US Senate during the period from 1979 to 2012. As we can observe, the slope of the Forman–Ricci network entropy is fairly flat during the first eight years (from 1979 to 1986), then it starts increasing with a significant slope up to the year 1994. After a short stagnation between 1994 and 1995, the Forman–Ricci network entropy regains in trend and increases at about the same pace up to the end of the observations in 2012. Figure 13 also shows the linear regression fit (marked as red dashed lines) for the two time periods 1986–1994 and 2000–2012 with slopes 0.0550 and 0.0424, respectively. The brief stagnation between 1994 and 1995 indicates a phase transition in the political network which was also observed in^3,34^.

**Figure 14:**
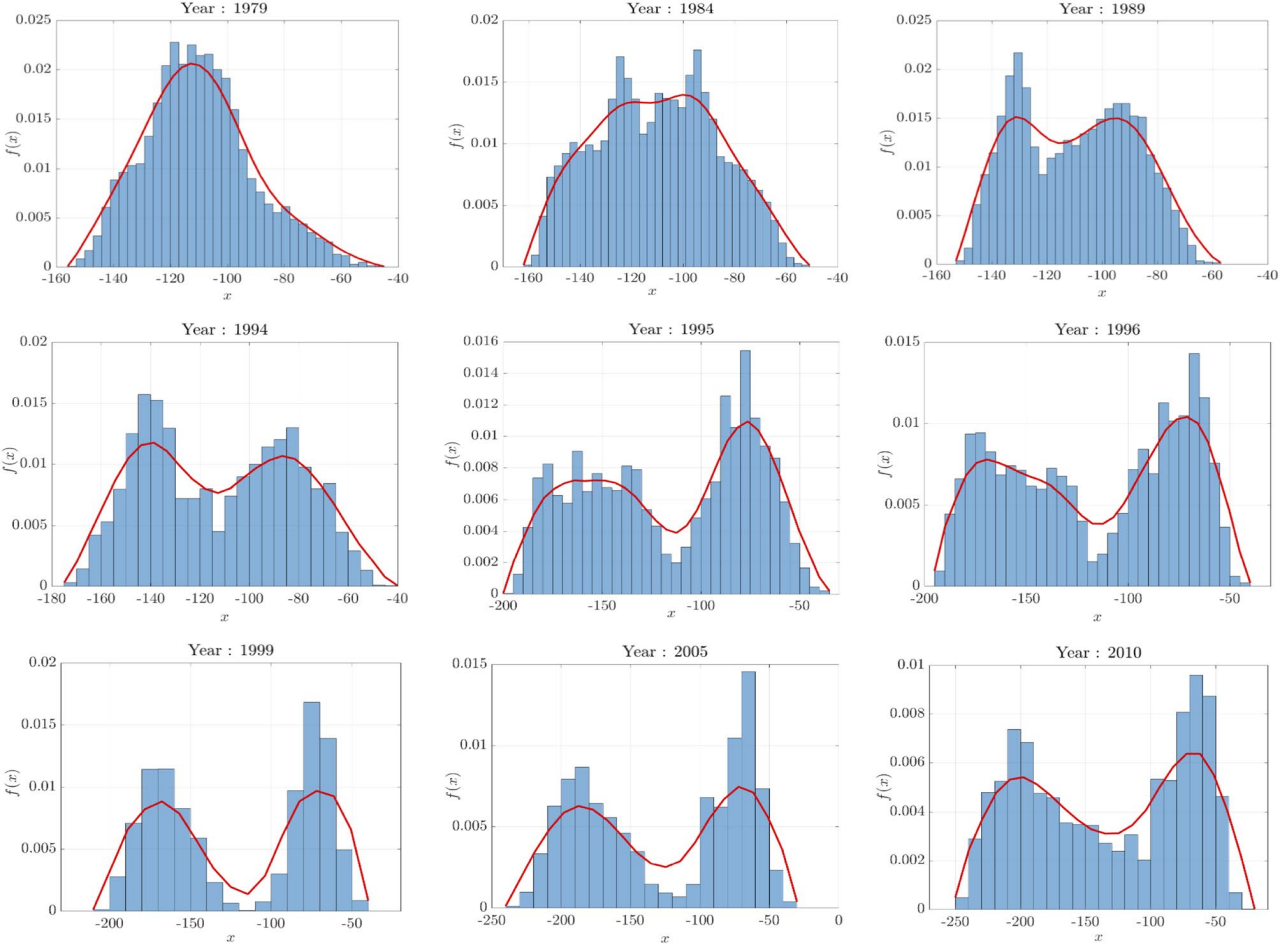
Forman–Ricci curvature distribution associated to networks constructed from the US Senate roll call dataset in different years. The Forman–Ricci distribution varies in time and starts exhibiting bimodal behavior after the year 1994.

We can also depict the phase transition by looking at the Forman-RC PDF for these TVCNs presented in Fig. 14. Here, we provide plots for the estimated Forman-RC distributions corresponding to networks constructed from the US Senate dataset in different years (1979, 1984, 1989,1994, 1995, 1996, 1999, 2005, 2010). We observe that the support of the Forman-RC distribution gets wider and evolves towards a bimodal distribution while the network evolves in time. Furthermore, we remark that the Forman-RC distribution starts exhibiting a bimodal behavior after the year 1994 which explains the phase transition. The reason for this phase transition is the 1994 election where Republicans capture the House of Representatives in the USA for the first time after 1956. This observation has also been argued in political science where they claim that the 1994 election was considered as the end of the conservative coalition. From a mathematical perspective, this result indicates that the network structural and dynamic properties encoded in the weights of the TVCNs change and obey different trends. Contrary to the density estimators used to plot Forman–Ricci curvature distributions in Figs. 2e and 3d, the error, in this case, is larger. This can be attributed to the network size considered for the US Senate dataset ($N = 100$).

### Discussion

Although modeling complex interactions is more or less feasible through CNs, it remains difficult to analyze and decipher whether these networks are about to experience a phase change especially when their generative model is unknown or when access to their evolution snapshots is limited. This task seems harder when it comes to the analysis of TVCNs, especially for understanding the properties of a time-varying network that extends beyond current sensing capabilities. The influence of leading social network companies, such as Facebook and Twitter, has already had far-reaching influence in people’s daily lives, ranging from significant changes like the way people consume media to seemingly simple things like getting informed about events that one’s circle of friends is attending. To achieve such goals and inspire advanced applications, more innovative approaches need to be investigated for analyzing TVCNs.

In this regard, in this paper, we have provided a novel geometric inspired framework for detecting phase changes in the topological properties of a CN, hence, a better understanding of the dynamics of such networks. This represents a crucial step towards designing sophisticated algorithms for prediction and control of their kinetics. Based on experimental studies, we have shown the effectiveness of the proposed Forman-RC algorithm to detect different states of a TVCN and determine the transition between the states accurately. In fact, we have tested our general framework on artificially generated networks based on different models, where the topology changes by varying the model parameters. Through an entropic measure of the Forman-RC distribution, we can detect the topological changes induced by the variation of the parameters. Unlike previous methods which rely on the network generating models or mechanisms, the Forman-RC approach is model agnostic.

The time complexity of this method is comparable to if not superior to most community detection methods when applied directly to each network snapshot just to find the phase transitions. The time complexity of computing the Forman-RC network entropy for one network snapshot is ${\mathscr {O}}(km)$ with $k \ll n$, where *k* is the average degree, *n* is the total number of nodes, and *m* is the total number of edges (e.g., see “Methods” section for a discussion on the time complexity). This is in contrast to modularity-based community identification approaches which require to find the community partition first. For example, the Newman’s spectral method for a sparse network has ${\mathscr {O}}(n^2\log n)$^35^ time complexity to calculate the maximum modularity partitions for each network snapshot. Alternatively, the Clauset, Newman and Moore (CNM) method which employs a greedy heuristic approach to find the maximum modularity partitions has ${\mathscr {O}}(n\log ^2 n)$ time complexity^36^; however, this may lead to results that differ from the optimal solution and the obtained modularity value is inaccurate compared to other methods^35^. Hierarchical agglomerative methods tend to have a time complexity of ${\mathscr {O}}(n^2 \log n)$. Most Bayesian (or semi-Bayesian) inference algorithms have either ${\mathscr {O}}(mb^2)$ or ${\mathscr {O}}(m)$ time complexities, where *b* is the number of communities. Notably, a non-parametric Bayesian approach achieves ${\mathscr {O}}(m)$ without explicitly dependent on the number of groups being inferred^37^.

Finally, we have also applied the developed curvature inspired algorithm to real-world datasets where we know a change point has been observed from previous experimental studies: one from biology where we detected a bifurcation point in human fibroblasts, and a second one from the roll call data of the US Senate, where we detected a change point after the 1994 election which marks the end of the conservative coalition.

We hope that this general framework, with the incorporation of geometry in the network analysis to detect its phase transitions, will receive more attention in other domains. Applications where the interaction between components is driving the dynamics of the network, such as social networks, neuronal activities^2^, and brain cognition are promising areas for the use of this framework.

## Methods

We consider an undirected time-varying weighted CN modeled as time-dependent graph $G_t=\{V_t, E_t\}$, where *t* is the time index, $V_t(G_t)$ designates the set of *N* nodes and $E_t$ represents the set of edges connecting them. The connection between two nodes *i* and *j*, denoted by $w_{ij}$, represents the interaction or similarity between them and it can be determined from a given dataset through information theoretic or artificial intelligence methods. Also, one can infer the interactions from the set of observations $x_1 (t),x_2 (t),\ldots ,x_N (t)$ where $x_i (t)$ describes the time evolution of node *i* in the network $G_t$. Our goal is to study how the local interactions, encoded in the graph’s adjacency matrix, change in time and then deduce the global behavior (i.e., the state) of the network under investigation.

Unlike existing work on studying CNs that analyze them using a node-based approach (e.g., node degree distribution), we focus on multiscale effects of CNs (i.e., how microscopic or local node to node interactions influence the overall macroscopic or global properties encoded in the geometry of networks). With this in mind, we develop a mathematical framework that takes into account the weights for analyzing the CN structure and retaining the maximum amount of information about the CN dynamics. Consequently, we compute the Forman-RC associated to each graph $G_t$ as is described next and investigate when the diversity in the curvature profile exhibits a change.

### Forman–Ricci curvature

Forman-RC is an edge-based approach that can identify the higher-order connectivity structure between different components in CNs^28^. Forman’s definition is inspired from the Bochner operator defined in differential geometry. The Forman-RC is computed for every edge in the graph, and its value is determined using an expression analogous to Bochner–Weitzenböc formula. For weighted CNs, the Forman-RC for an existing edge *e* between node *i* and node *j* in a given graph is calculated as follows:

1 $$\begin{aligned} FR(e)=w(e) \left( \frac{w(i)}{w(e)} - \sum \limits _{e_i\sim e} \frac{w(i)}{\sqrt{w(e)w(e_i)}} + \frac{w(j)}{w(e)}- \sum \limits _{e_j\sim e} \frac{w(j)}{\sqrt{w(e)w(e_j)}}\right) , \end{aligned}$$

where *w*(*e*) is the weight for the edge *e*, $e_i \sim e$ and $e_j \sim e$ are all the edges except edge *e* connected to nodes *i* and *j*, respectively, and *w*(*i*) and *w*(*j*) are the weights of nodes *i* and *j*, respectively. Equation (1) shows that the Forman-RC depends implicitly on node degrees, but it also takes into account the edge weights (node-to-node interactions) which helps us to deduce the dominant nodes linked to a node *i* by investigating the curvature of each edge connected to it. Consequently, the time complexity for the edge Forman-RC computation is linear ${\mathscr {O}}(k)$ with $k \ll n$, where *k* is the average degree, and *n* is the total number of nodes. Therefore, we believe that this geometric interpretation is a key step to effectively analyze a large CN and better understand its topology.

Consequently, we form a new weighted graph at each time where the weights represent the curvature coefficients associated with each edge in the original TVCN. This new graph is a new representation of the topological properties of the given graphs that encapsulates non-trivial characteristics of the network (the curvature of the network components). Hence, such a new time-dependent topological representation of the CN allows us to understand the causal interdependence between nodes better and to quantify the information propagation between major components in CN across time.

Inspired by statistical mechanics and thermodynamics, where the states of a given system are closely related to its entropy (i.e., Boltzmann entropy), we link the states of a given network to an entropic measure. The entropy can be considered as a measure of the diversity or disorder of a given system (Boltzmann) or the lack of information about it which has also been quantified by Shannon entropy that is used in information theory^38^. Here, we measure the state of the CN $G_t$ by computing the “*Forman–Ricci network entropy*” that we define as the Shannon entropy of the Forman-RC of the CN, i.e.,

2 $$\begin{aligned} H_R(G_t)=-\int f_{RC}(x)\log _2(f_{RC}(x)) dx, \end{aligned}$$

where $f_{RC}(\cdot )$ is the probability density function (PDF) corresponding to the random variable Forman-RC in the network $G_t$. The aforementioned probability density function is estimated using a non-parametric wavelet-based approach^39^ (more details are provided in the next paragraph). The goal is to use the Forman–Ricci network entropy as a metric that will identify the states of the TVCN.

### Wavelet-based estimator for Forman–Ricci curvature distribution

As discussed in our algorithmic strategy for detecting phase changes, we have to estimate the probability density function (PDF) corresponding to the Forman-RC curvature in a network $G_t$. For the sake of accuracy, we employ a wavelet-based approach to estimate the PDF and then to evaluate the Forman–Ricci network entropy. Next, we provide a brief description of the aforementioned approach^39–46^. Let *f*(*x*) be the PDF which we wish to estimate from *N* given samples of the Forman–Ricci curvature of edges $\{x_i\}_{i=1}^N$ . The fundamental idea of wavelets is based on the Fourier analysis and expresses the PDF as a series expansion in a set of functions. Initially, we choose two functions, $\varphi (x)$ and $\psi (x)$ called scaling and wavelet function, respectively, which satisfy certain orthogonality properties. Then, by translating and scaling the aforementioned functions, we construct the families of functions $\{\varphi _{j,n}(x) = 2^{\frac{j}{2}}\varphi (2^j x -n)\}_{n\in {\mathbb {Z}}}$ and $\{\psi _{j,n}(x) = 2^{\frac{j}{2}}\psi (2^j x -n)\}_{n\in {\mathbb {Z}}}$, where *j* is an integer parameter that controls the accuracy of the approximation. Then, we express the PDF as follows:

3 $$\begin{aligned} f(x) = \sum _{n\in {\mathbb {Z}}} \left( f_{j,n}\varphi _{j,n}(x) + \sum _{s=j}^{j_1} \tilde{f}_{s,n}\psi _{s,n}(x) \right) , \end{aligned}$$

where $j_1$ is determined by the number of data samples, $f_{j,n} = \int _{{\mathbb {R}}} f(x) \varphi _{j,n}(x) dx$ and $\tilde{f}_{j,n} = \int _{{\mathbb {R}}} f(x) \psi _{j,n}(x) dx$ designate the scaling and wavelets coefficients, respectively. Since *f*(*x*) is a PDF, then the coefficients $f_{j,n}$ and $\tilde{f}_{j,n}$ can be interpreted as the expected value over the samples of the scaling function and the wavelet function, respectively. Hence, the coefficients can be calculated from the given samples as follows:

4 $$\begin{aligned} f_{j,n} = \frac{1}{N}\sum _{i=1}^N \varphi _{j,n}(x_i), \mathrm {~and~} \tilde{f}_{j,n} = \frac{1}{N}\sum _{i=1}^N \psi _{j,n}(x_i). \end{aligned}$$

Therefore, the coefficients of Eq. (3) are calculated by averaging the chosen scaling and wavelet functions evaluated on the given samples. There are several families of scaling and wavelet functions and in practice one chooses the family based on the expected complexity of the PDF to be estimated. In our framework, we use the Daubechies family with parameter 4 (generally denoted as db4).

### Transition boundary quantification

As illustrated in the random growing networks example (see Fig. 2), providing an accurate estimation of the phase transition boundaries is not trivial. Here, we propose to introduce a hyper-parameter that depends on the network size $\gamma _N$ that defines the boundaries $\alpha _1$ and $\alpha _2$ that separates the first phase from the second phase and the second phase from the third phase, respectively. Given the parameter $\gamma _N$, we define the interval $\textrm{I}$ as $\textrm{I} = \{\alpha _t \ge 0 ~ | ~ \left| \frac{dH_R(\alpha _t)}{d\alpha _t}\right| \le \gamma _N \}$, and the boundaries are defined as follows:

5 $$\begin{aligned} \alpha _1 = \mathop {{{\,\mathrm{arg\,max}\,}}}\limits _{\alpha _t \in [0,1] \cap ~\textrm{I}} \left| \frac{dH_R(\alpha _t)}{d\alpha _t}\right| , \quad \text {and}\quad \alpha _2 = \mathop {{{\,\mathrm{arg\,min}\,}}}\limits _{\alpha _t \in [1, +\infty ) \cap ~\textrm{I}} \left| \frac{dH_R(\alpha _t)}{d\alpha _t}\right| , \end{aligned}$$

The choice of the hyper-parameter must be a function of the peak of the first derivative of the entropy associated with the Forman–Ricci curvature values of a network (which is indeed a function of the network size). Therefore, we can choose the parameter $\gamma _N$ to be proportional to the peak value. Note that the coefficient of proportionality depends on the application and fixed by the user. As an example, for the random growing network study with $N = 10{,}000$ nodes, we remark that the peak is large so we choose $\gamma _N = \frac{1}{5} \times \max _{\alpha _t}\frac{dH_R(\alpha _t)}{d\alpha _t}$ and we get $\alpha _1 = 0.6$ and $\alpha = 1.45$. However, we have to choose a smaller coefficient for smaller network sizes.

### Time complexity

The overall time complexity of the proposed algorithm can be broken down as follows: (1) the Forman-RC curvature calculation for one network snapshot is ${\mathscr {O}}(km)$, (2) the density estimation is ${\mathscr {O}}(\ell m)$, and (3) the entropy computation is ${\mathscr {O}}(\ell )$ with $k \ll n$ and $\ell \ll m$ where *k* is the average degree, *n* is the number of nodes, *m* is the number of edges, $\ell$ is the number of sample points of the density from the binning procedure. Therefore, the time complexity for one network snapshot computation of the entropy associated with the Forman-RC curvature of a network is ${\mathscr {O}}(km) + {\mathscr {O}}(\ell m)$.

### Preprocessing of the weights in the artificial neural networks experiment

During the training phase, the weights associated with each epoch can assume a range of real values based on the gradient. However, in order to compute and evaluate the Forman–Ricci curvature for each edge, it is necessary for the weights to be positive. To address this requirement, we employ the Min-Max normalization technique for each epoch, which scales the weight values to a range between 0 and 1.

### Preprocessing of the roll call data of the US senate

The preprocessing task for the data is done in two steps. First, we take the records of the 100 Senate members, then the “*yes*” and “*no*” decisions are mapped to 1 and 0, respectively. Since the Senate members cannot be the same during the entire period (1979–2012), we solved this issue in a similar way as the technique used by the authors in^3^. We look at the record of the votes per seat and not per Senate member. For missing values, we insert a value of 1 or 0 corresponding to the member’s party decision obtained by majority polling on that particular vote. The result of the first step is an unweighted matrix of size 7949 (i.e., the entries are zeros and ones designating disagreements ans agreements, respectively). The second step consists of constructing a probabilistic measure that encodes the probability of having an agreement between any two given nodes (i.e., Senate seat). To determine the probability between two nodes *i* and *j*, we compute the average matrix of a block of matrices (votes). Since we are analyzing the votes for 33 years from 1979 to 2012, we choose the size of the block to be equal to the average annual votes $7949/33 \approx 240$. Therefore, by computing the average matrix of every 240 votes we get 33 networks encoding the annual probability of agreement between Senate seats during the entire period of study.

## Data Availability

The datasets used and/or analyzed during the current study are available from the corresponding author on reasonable request.
